# Loss of cytoplasmic incompatibility in *Wolbachia*-infected *Aedes aegypti* under field conditions

**DOI:** 10.1371/journal.pntd.0007357

**Published:** 2019-04-19

**Authors:** Perran A. Ross, Scott A. Ritchie, Jason K. Axford, Ary A. Hoffmann

**Affiliations:** 1 Pest and Environmental Adaptation Research Group, Bio21 Institute and the School of BioSciences, The University of Melbourne, Parkville, Victoria, Australia; 2 College of Public Health, Medical and Veterinary Sciences, James Cook University, Smithfield, Queensland, Australia; 3 Australian Institute of Tropical Health and Medicine, James Cook University, Smithfield, Queensland, Australia; The Pennsylvania State University, UNITED STATES

## Abstract

*Wolbachia* bacteria are now being introduced into *Aedes aegypti* mosquito populations for dengue control. When *Wolbachia* infections are at a high frequency, they influence the local transmission of dengue by direct virus blocking as well as deleterious effects on vector mosquito populations. However, the effectiveness of this strategy could be influenced by environmental temperatures that decrease *Wolbachia* density, thereby reducing the ability of *Wolbachia* to invade and persist in the population and block viruses. We reared *w*Mel-infected *Ae*. *aegypti* larvae in the field during the wet season in Cairns, North Queensland. Containers placed in the shade produced mosquitoes with a high *Wolbachia* density and little impact on cytoplasmic incompatibility. However, in 50% shade where temperatures reached 39°C during the day, *w*Mel-infected males partially lost their ability to induce cytoplasmic incompatibility and females had greatly reduced egg hatch when crossed to infected males. In a second experiment under somewhat hotter conditions (>40°C in 50% shade), field-reared *w*Mel-infected females had their egg hatch reduced to 25% when crossed to field-reared *w*Mel-infected males. *Wolbachia* density was reduced in 50% shade for both sexes in both experiments, with some mosquitoes cleared of their *Wolbachia* infections entirely. To investigate the critical temperature range for the loss of *Wolbachia* infections, we held *Ae*. *aegypti* eggs in thermocyclers for one week at a range of cyclical temperatures. Adult *w*Mel density declined when eggs were held at 26–36°C or above with complete loss at 30–40°C, while the density of *w*AlbB remained high until temperatures were lethal. These findings suggest that high temperature effects on *Wolbachia* are potentially substantial when breeding containers are exposed to partial sunlight but not shade. Heat stress could reduce the ability of *Wolbachia* infections to invade mosquito populations in some locations and may compromise the ability of *Wolbachia* to block virus transmission in the field. Temperature effects may also have an ecological impact on mosquito populations given that a proportion of the population becomes self-incompatible.

## Author summary

*Aedes aegypti* mosquitoes infected with *Wolbachia* symbionts are being deployed in the tropics as a way of reducing disease transmission. Some *Wolbachia* strains are vulnerable to high temperatures but these effects have not been evaluated outside of a laboratory setting. We reared *Ae*. *aegypti* infected with the *w*Mel strain of *Wolbachia* in the field during the wet season in Cairns, Australia, where the first releases of *Wolbachia*-infected *Ae*. *aegypti* took place. *w*Mel-infected mosquitoes became partially self-incompatible, with reduced egg hatch, when larvae were reared in partial shade where maximum daily temperatures exceeded 39°C. Under these conditions the amount of *Wolbachia* in adult mosquitoes was reduced to less than 1% of laboratory-reared mosquitoes on average, while some mosquitoes were cleared of *Wolbachia* entirely. In contrast, *w*Mel was stable when mosquitoes were reared under cooler conditions in full shade. Field trials with the *w*Mel strain are now underway in over 10 countries, but high temperatures in some locales may constrain the ability of *Wolbachia* to invade natural mosquito populations and block disease transmission.

## Introduction

*Aedes aegypti* mosquitoes are the principal vectors of dengue and are widespread in the tropics where they live near humans [1, 2]. Chemical insecticides have historically been used to control *Ae*. *aegypti* populations during disease outbreaks, but this approach is unlikely to be sustainable as insecticide resistance is now widespread in many parts of the world [3, 4]. There is increasing interest in ‘rear and release’ programs where mosquitoes modified with desirable traits are released into natural populations as an alternative approach to disease control [5]. At the forefront of these programs is the deployment of mosquitoes infected with *Wolbachia* bacteria. *Wolbachia* occur naturally in many insects but have been introduced experimentally into *Ae*. *aegypti* where they can interfere with the transmission of dengue and other pathogens [6–8]. *Wolbachia* are transmitted maternally and typically affect host reproduction [9] or provide other advantages [10] to facilitate their spread into populations. These phenotypes have been utilized in disease control programs where *Wolbachia*-infected mosquitoes have been deployed to replace natural populations [11–13] or suppress populations through the release of only males [14, 15]. Both approaches rely on cytoplasmic incompatibility induced by *Wolbachia*, where uninfected females that mate with infected males do not produce viable offspring, but viability is restored if the female is also *Wolbachia*-infected [16, 17].

Over ten *Wolbachia* strain associations have now been generated in *Ae*. *aegypti* and they exhibit a diverse range of phenotypes. Some *Wolbachia* strains are relatively benign and have little impact on host fitness or virus blockage such as the *w*Ri strain [18]. Others impose large fitness costs but also strongly reduce virus transmission including *w*MelPop [17] and *w*Au [19] while others like *w*AlbB fall somewhere in between [20, 21]. There are also superinfections where two or more *Wolbachia* strains infect the same host, which can have combined or unexpected effects [21, 22]. *Aedes aegypti* infected with the *w*Mel strain of *Wolbachia* have been or are now being released in over ten countries (https://www.worldmosquitoprogram.org/) and have successfully established in suburban areas in Cairns and Townsville in Queensland, Australia [11–13] and in Brazil [23]. The *w*AlbB strain has also been deployed successfully in Malaysia for population replacement (http://www.imr.gov.my/wolbachia/) and in several countries for population suppression, where the release of only infected males has reduced population sizes by more than 80% due to cytoplasmic incompatibility (https://debug.com/; https://www.nea.gov.sg/corporate-functions/resources/research/wolbachia-aedes-mosquito-suppression-strategy).

Despite these successes, there are limitations of *Wolbachia* infections that may affect their utility as disease control agents (reviewed in Ritchie et al. [24]). The majority of *Wolbachia* infections in *Ae*. *aegypti* reduce mosquito fitness and these costs tend to be exacerbated in stressful environments such as when larvae are starved [25] or in quiescent eggs [18, 19, 26, 27]. Fitness costs can have enormous effects on invasion success. For example, the *w*MelPop infection failed to persist in release zones in Australia and Vietnam despite reaching frequencies above 90%, likely due to the massive fitness costs of this strain [28]. *Wolbachia* infections that occur naturally in mosquitoes can interfere with patterns of cytoplasmic incompatibility and limit the potential for population replacement and suppression [29]. Density-dependent interactions [30] and spatially heterogeneous environments [31] can also slow the rate of invasion, as can pesticide susceptibility in released mosquitoes [23].

For population replacement programs to be successful, *Wolbachia* infections should persist at high frequencies in the environment and block virus transmission under field conditions for many years following deployment [24]. There is a risk that *Wolbachia* infections, viruses or mosquitoes will evolve following the establishment of *Wolbachia* in populations, leading to less effective virus protection in *Wolbachia*-infected mosquitoes in the long-term [32]. However, the *w*Mel infection has remained stable so far in terms of virus blockage [33] and its effects on fitness [34]. After seven years in the field, *w*Mel has retained a high titer and continues to induce complete cytoplasmic incompatibility in the laboratory [35], indicating that attenuation is unlikely for at least several years following deployment.

While the *w*Mel infection in northern Queensland *Ae*. *aegypti* populations does not appear to have changed phenotypically since release, environmental conditions in the field such as temperature can have transient effects on *Wolbachia* infections, influencing their ability to suppress virus transmission or establish in populations. This issue is particularly important as climate change is leading to warmer average conditions and higher temperature extremes, including in the tropics [36]. *Wolbachia* infections in *Ae*. *aegypti* are vulnerable to high temperatures; heat stress during larval development reduces *Wolbachia* density in adults [37] and decreases the fidelity of cytoplasmic incompatibility and maternal transmission [35, 38]. The fidelity of cytoplasmic incompatibility and maternal transmission are two key parameters for *Wolbachia* spread [39] while *Wolbachia* density is positively associated with the strength of virus blockage in both *Drosophila* [40, 41] and mosquitoes [42].

*Wolbachia* strains in *Ae*. *aegypti* differ in their response to heat stress; the *w*Mel and *w*MelPop strains are relatively susceptible while *w*AlbA, *w*AlbB and *w*Au are more robust, retaining high densities when larvae are reared at cyclical temperatures of 26–37°C [19, 38]. These laboratory studies demonstrate the potential for heat stress to affect the success of *Wolbachia* interventions, but conditions experienced by mosquitoes in field situations are more complex than in an incubator. To understand the effects of high temperatures under more natural conditions, we reared *Ae*. *aegypti* larvae infected with *w*Mel in the field with varying levels of exposure to sunlight. We then performed crosses to test for effects on cytoplasmic incompatibility and measured *Wolbachia* density. Finally, we performed experiments with *Ae*. *aegypti* eggs in the laboratory to determine the range of temperatures that adversely affect *Wolbachia* infections.

## Methods

### Ethics statement

Blood feeding on human subjects was approved by the Human Research Ethics Committee, James Cook University (approval H4907). All adult subjects provided informed oral consent (no children were involved).

### Mosquito strains and colony maintenance

*Aedes aegypti* mosquitoes infected with the *w*Mel strain of *Wolbachia* were collected in 2013 from locations near Cairns, Australia where *w*Mel had successfully established [11]. *Aedes aegypti* infected with *w*MelPop were collected from Cairns, Australia in 2012 following field releases and local field breeding [28]. *Aedes aegypti* infected with *w*AlbB were derived from laboratory colonies and are described in Xi et al. [16] and Axford et al. [20]. Uninfected *Ae*. *aegypti* were collected in 2016 from locations where *Wolbachia*-infected mosquitoes had not been released. Mosquitoes were maintained in an insectary at the University of Melbourne according to methods described by Ross et al. [43]. Females with each *Wolbachia* strain were crossed to males from the uninfected population for at least three generations prior to the start of experiments to ensure that genetic backgrounds between populations were similar [27].

### Larval rearing under field temperature conditions

We conducted two experiments during the wet season in Cairns, Australia in 2018 to test the stability of the *w*Mel *Wolbachia* infection under field temperature conditions. Experiments took place at James Cook University under a protective awning with different levels of shade as described in Ritchie et al. [44]. The relative reduction in light intensity compared to direct sunlight was determined using an EasyView EA30 light meter (Extech Instruments Corporation, Waltham, MA 02451 U.S.A.); two shade levels were chosen for the experiments which reduced light intensity by 50% and 99% respectively. We then established water-filled containers of various sizes to simulate a range of field larval habitats. The *w*Mel-infected eggs were hatched in a single tray in 99% shade, then approximately 100 1^st^ instar larvae were placed into each container. Larvae were provided with TetraMin tropical fish food tablets (Tetra, Melle, Germany) *ad libitum* throughout their development. Water temperatures were recorded every 30 minutes for the duration of larval development with data loggers (Thermochron; 1-Wire, iButton.com, Dallas Semiconductors, Sunnyvale, CA, USA) placed in zip-lock bags and submerged in the centre of each container. Pupae were collected one week after hatching and were returned to the laboratory for adult emergence.

In the first experiment, *Ae*. *aegypti* eggs infected with the *w*Mel strain of *Wolbachia* were hatched on the 10^th^ of January 2018 and pupae were collected on the 17^th^ of January. We used containers of three types with a wide range of water volumes which were expected to experience a variety of temperature conditions. Black buckets (13 cm radius, 25 cm height) were filled with 8 L of tap water, plant pots (5 cm radius, 10 cm height) with 500 mL and cups (2 cm radius, 6 cm height) with 60 mL. Containers were covered with mesh or stockings to prevent wild mosquitoes from ovipositing, and experimental mosquitoes from escaping. This setup was repeated for both the 99% and 50% shade levels. *w*Mel-infected larvae were also reared in a single bucket filled with 8 L of water and placed in direct sunlight.

In the second experiment, we repeated this procedure but used two container types: black buckets filled with 8 L of water and small round clear plastic containers (10 cm radius, 7 cm height) filled with 400 mL of water, with each container replicated three times at 99% and 50% shade levels. Eggs were hatched on the 26^th^ of January 2018 and pupae were returned to the laboratory on the 1^st^ of February. Populations of *w*Mel-infected and uninfected *Ae*. *aegypti* were also reared in the laboratory concurrently at 26°C ± 1°C according to Ross et al. [43] for experimental crosses.

### Cytoplasmic incompatibility after field rearing

We tested the ability of *w*Mel-infected males to induce cytoplasmic incompatibility and *w*Mel-infected females to restore compatibility after being reared under field temperature conditions. Adults emerging from each container type and shade level were added to 15 cm^3^ cages (BugDorm-4M1515, Megaview Science Co., Taichung, Taiwan) where sexes were maintained separately. Crosses were performed two to three days after adults emerged by aspirating approximately 50 females into cages with an equal number of males. Females were blood fed three days later and then isolated in plastic cups containing 15 mL of larval rearing water and a strip of sandpaper for oviposition. Eggs were collected four days after blood feeding, partially dried, and then hatched four days after collection. Eggs were then counted under a dissecting microscope and hatch rates were determined by counting the proportion of eggs that had a clearly detached cap.

In the first experiment, we performed crosses with adults reared in buckets at either 99% or 50% shade. Field-reared *w*Mel-infected males were crossed to uninfected females to determine their ability to induce cytoplasmic incompatibility. We also crossed field-reared *w*Mel-infected females to either uninfected males or *w*Mel-infected and laboratory-reared males to determine the ability of females to restore compatibility. Infected males and uninfected females both reared under laboratory conditions were crossed to each other to confirm that *w*Mel induces complete cytoplasmic incompatibility at 26°C. Twenty females were isolated for oviposition in each of these crosses, but individuals that died or did not lay eggs were excluded from the analysis.

In the second experiment we performed a similar set of crosses but only for adults emerging from buckets held in 50% shade. In crosses where both sexes were infected we used males and females from the same container rather than using males reared at 26°C. This was done to see if populations became self-incompatible when both sexes were reared at warmer temperatures. Thirty females were isolated for oviposition in each cross. Crosses between males and females reared under the same conditions were also performed with adults that were reared in buckets held in 99% shade and small containers held at 50% and 99% shade. We determined egg hatch proportions and *Wolbachia* densities of females in each of these crosses to see if there was a relationship between *Wolbachia* density and hatch rate.

### Egg thermal tolerance and *Wolbachia* density

We performed two experiments using thermocyclers (Biometra, Göttingen, Germany) to test the thermal tolerance of *Wolbachia*-infected Ae. *aegypti* eggs and the density of *Wolbachia* under a range of temperature conditions. We followed methods described in Kong et al. [45] with some modifications. Eggs from uninfected, *w*Mel, *w*AlbB and *w*MelPop colonies were collected on sandpaper strips which were then partially dried, wrapped in paper towel and held in sealed zip-lock bags. Four days after collection, eggs were brushed onto filter paper with a small paint brush and then tipped into 0.2 mL PCR tubes using a funnel. Batches of 15 to 39 eggs (mean 25.7) were added to each tube. Tubes were closed and then tapped on the bench to ensure that eggs sank to the bottom of the tube where temperature control in the thermocycler is the most accurate [45]. Tubes were then placed in heat blocks of Biometra TProfessional TRIO 48 thermocyclers with tubes from each population arranged randomly in each block.

In both experiments we used three thermocyclers, each with three heated blocks that can run independently for a total of 9 temperature regimes. In the first experiment we chose a broad range of temperature cycles to cover the entire range of temperatures that *Ae*. *aegypti* may experience in the field (http://www.bom.gov.au/climate/averages/tables/cw_031011_All.shtml). Each regime had a fluctuation of 10°C between the minimum and maximum temperature; the lowest being 8–18°C and the highest being 32–42°C, with a difference of 3°C between each regime (S3A Fig). In the second experiment we chose a narrower temperature range based on when egg hatch and *Wolbachia* density started to decline in the previous experiment. The lowest regime was set to 24–34°C and the highest was 32–42°C, with difference of 1°C between each regime (S3B Fig). In the first experiment there were six replicate tubes of eggs for each temperature cycle and *Wolbachia* infection type and the second experiment had 12 replicates. After all tubes were added to the thermocyclers we closed the lids and started programs simultaneously. Eggs in tubes were also maintained at 26°C in a controlled temperature room in both experiments.

After one week, tubes were removed from the thermocyclers and eggs were hatched by holding PCR tubes sideways above 70 mL specimen cups and then pipetting water into the tubes so that eggs fell into the cup. Each cup was filled with 40 mL of water and provided with a small amount of TetraMin and a few grains of yeast. Two days after hatching we determined egg hatch proportions by dividing the number of larvae by the number of eggs. We counted larvae again every 2 days as some eggs were slow to hatch, allowing one week in total for larvae to appear before we ceased counting. All larvae that hatched were added to plastic containers filled with 500mL of RO water and reared to adulthood. Multiple replicate cups of larvae were combined into trays for rearing, but the larval density was controlled to 100 larvae per tray or fewer to account for effects of larval competition and development time on *Wolbachia* density [46]. All adults were stored in ethanol for *Wolbachia* density measurements.

### *Wolbachia* detection and density

In each experiment, random subsets of adults were stored in ethanol within 24 hours of emergence for *Wolbachia* screening. For both field experiments we extracted DNA from 16 males and 16 females from each container type and shade level. For experiments with eggs held in thermocyclers we extracted DNA from up to 10 (first experiment) or 12 (second experiment) males and 10 or 12 females from each *Wolbachia* infection type and treatment. Some treatments had lower sample sizes due to low egg hatch proportions. DNA was extracted from whole adults with 150 μL of 5% Chelex 100 resin (Bio-Rad Laboratories, Hercules, CA) and 3 μL of Proteinase K. We then conducted qPCR to detect and estimate the density of *Wolbachia* in each whole adult using methods described previously [47]. Individuals were considered uninfected if the *Ae*. *aegypti*-specific marker amplified successfully (Cp value < 35) but the *Wolbachia*-specific marker did not (Cp value of 35 or no Cp value) in two independent runs. For individuals that were positive for *Wolbachia*, (Cp value < 35 for both markers), differences in Cp between the two markers were transformed by 2^n^ to provide an estimate of *Wolbachia* density, averaged from at least two independent runs. For the second field experiment, we also estimated the *Wolbachia* density of females after they had laid eggs to see if there was a relationship between *Wolbachia* density and egg hatch rate when crossed to infected males reared under the same conditions.

### Statistical analysis

All data were analyzed using SPSS statistics version 24.0 for Windows (SPSS Inc, Chicago, IL). Hatch proportion and *Wolbachia* density data were often not normally distributed, and we therefore compared treatments for these variables with Kruskal-Wallis and Mann-Whitney U tests. We also used Spearman’s rank-order correlation to test the relationship between egg hatch and female *Wolbachia* density.

## Results

### First field experiment

**Fig 1:**
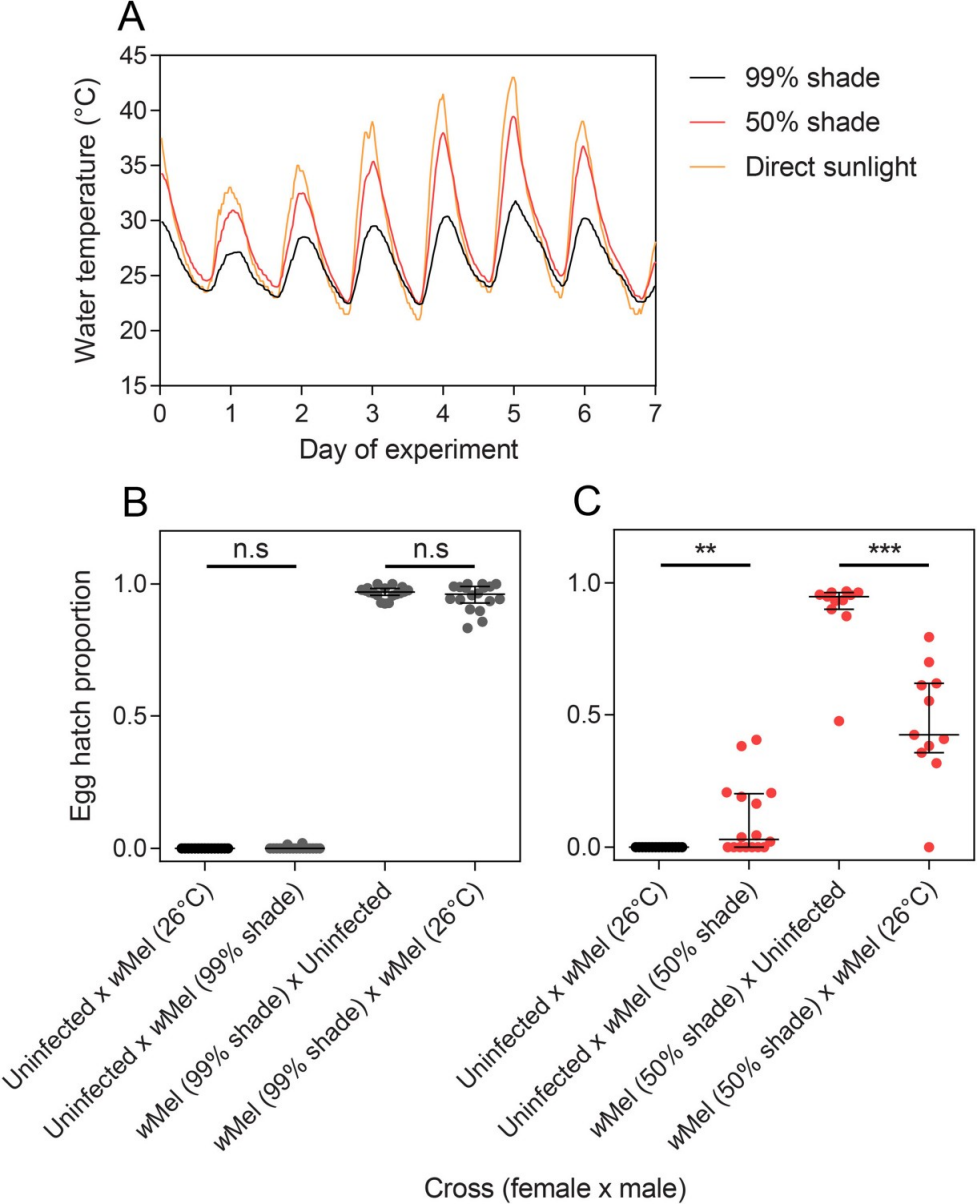
Temperature cycles and the resulting egg hatch proportions from crosses in the first field experiment. (A) Temperature cycles experienced in buckets (8 L) from the 10^th^ to the 17^th^ of January 2018. (B-C) Egg hatch proportions of crosses with *w*Mel-infected adults when larvae were reared under 99% shade (B) or 50% shade (C) in buckets. All error bars are medians with interquartile ranges. ** P < 0.01, *** P < 0.001 by Mann-Whitney U test.

We monitored water temperatures experienced by larvae in each container type at different levels of shade. Maximum temperatures differed between container types at 99% shade, with cups having average maximum daily temperatures that were 2.5°C higher than buckets, though average temperatures were similar because smaller containers reached cooler temperatures at night (S1 Fig). Temperature cycles were similar between containers in 50% shade, which was unexpected given the large differences in water volume. The level of shade affected temperature substantially, with buckets in direct sunlight experiencing average maximum temperatures of 38.3°C, while containers in 50% shade (average minimum: 23.7°C, average maximum: 35.3°C) were much warmer than containers in 99% shade (23.2–29.6°C) (Fig 1A).

We tested the ability of *w*Mel-infected males reared under field temperature conditions to induce cytoplasmic incompatibility with uninfected females. In a control cross, *w*Mel-infected males reared in the laboratory at 26°C caused complete cytoplasmic incompatibility (no eggs hatched) with uninfected females (Fig 1). *w*Mel-infected males reared in buckets at 99% shade induced almost complete cytoplasmic incompatibility, though 2/18 females produced a single viable progeny each (Fig 1B). *w*Mel-infected males reared in buckets at 50% shade induced weaker cytoplasmic incompatibility, with 9/16 females producing some viable progeny (Fig 1C).

We also tested the ability of *w*Mel-infected females to retain their compatibility with *w*Mel-infected males reared in the laboratory. When females were reared in buckets at 99% shade, there was no difference in hatch rate between crosses with uninfected males and crosses with *w*Mel-infected males (Mann-Whitney U: Z = 0.348, P = 0.726, Fig 1B). In contrast, *w*Mel-infected females reared in buckets at 50% shade had a 47.6% reduction in egg hatch rate when crossed to *w*Mel-infected males (Z = 3.612, P < 0.001). This indicates partial incompatibility with *Wolbachia*-infected males, suggesting a substantial loss of *Wolbachia* infection.

**Fig 2:**
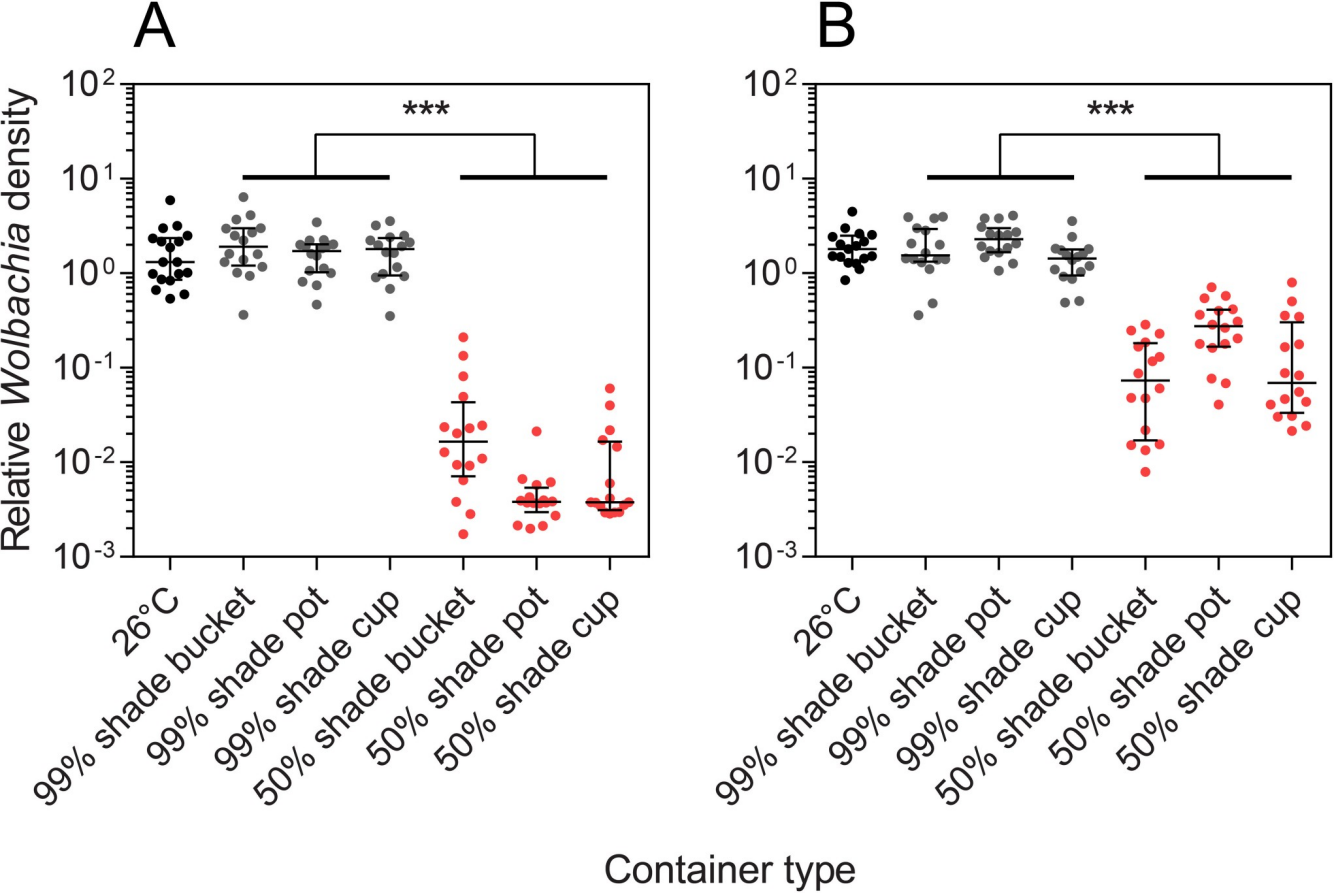
***Wolbachia* density of (A) Females and (B) males reared in different container types at two levels of shade in the first field experiment.** All error bars are medians with interquartile ranges. *** P < 0.001 by Kruskal-Wallis test.

We estimated the *Wolbachia* density of a subset of adults from each container type and level of shade (Fig 2). *Wolbachia* density was not consistently affected by container type for both females (Kruskal-Wallis χ^2^ = 2.598, df = 2, P = 0.273) and males (χ^2^ = 4.419, df = 2, P = 0.110), likely because the container types experienced similar temperature fluctuations at 50% shade. Conversely, *Wolbachia* density was affected substantially by shade level for both females (χ^2^ = 71.261, df = 1, P < 0.001) and males (χ^2^ = 68.563, df = 1, p < 0.001). Females reared under 50% shade had a median *Wolbachia* density that was just 0.32% of the laboratory control, while males had a density of 8.09% of the control. This reduction likely reflects the substantially higher maximum daily temperatures experienced in containers at 50% shade. In contrast, the *Wolbachia* density of adults reared at 99% shade was not significantly different to laboratory-reared adults (females: χ^2^ = 0.650, df = 1, P = 0.420, males: χ^2^ = 0.085, df = 1, P = 0.771).

All adults screened from containers in 99% shade, 50% shade and the laboratory were positive for *Wolbachia*. However, we were unable to detect any *Wolbachia* infection in a sample of 11 adults taken from a bucket placed in direct sunlight. This indicates a complete loss of infection which is likely due to the extreme temperatures experienced in that container (up to 43°C, Fig 1A). Though we did not score survival to adulthood in containers directly, the bucket placed in direct sunlight experienced high mortality since only 11 adults emerged out of the approximately 100 larvae added initially.

### Second field experiment

**Fig 3:**
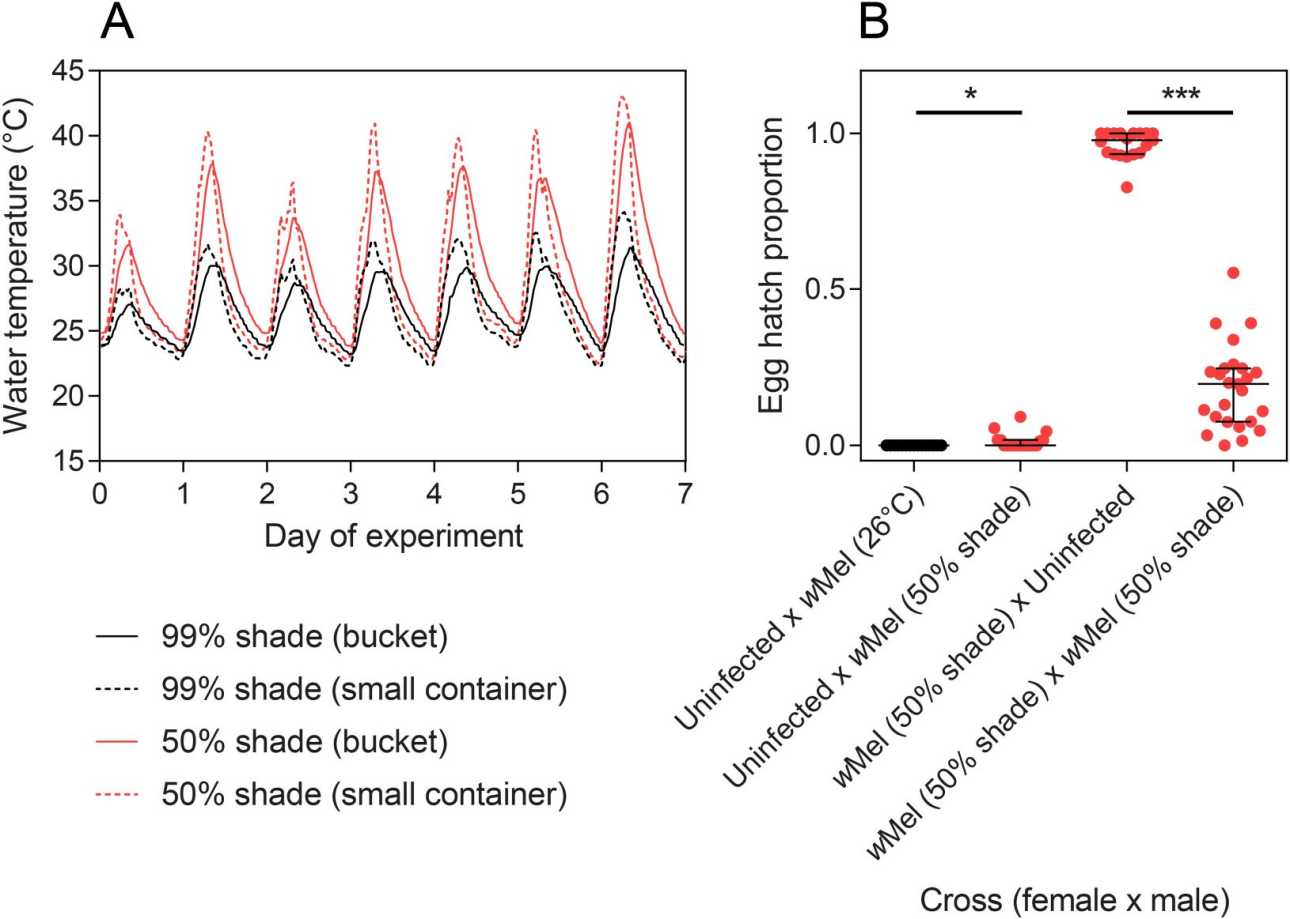
Temperature cycles and the resulting egg hatch proportions from crosses in the second field experiment. (A) Temperature cycles experienced in buckets (8 L) and small containers (400 mL) from the 26^th^ of January to the 1^st^ of February 2018. (B) Egg hatch proportions of crosses with *w*Mel-infected adults when larvae were reared in buckets under 50% shade. All error bars are medians with interquartile ranges. * P < 0.05, *** P < 0.001 by Mann-Whitney U test.

We conducted a second experiment later in the month where we also tested cytoplasmic incompatibility and measured *Wolbachia* density in adults. Temperatures were affected substantially by the location of containers where average maximum temperatures were nearly 7°C warmer in 50% shade compared to 99% shade (Fig 3A). Maximum temperatures also differed between container types; at 50% shade small containers reached 39.26°C on average while buckets reached 36.54°C, but average temperatures did not differ much between containers at the same level of shade because of warmer minimum temperatures in buckets.

We set up crosses with adults emerging from buckets held in 50% shade to test for any effects on cytoplasmic incompatibility. *w*Mel-infected males induced strong but incomplete cytoplasmic incompatibility with uninfected females; 7/18 females produced some viable progeny, compared to 0/20 in the control (Fig 3B). *w*Mel-infected adults reared in buckets at 50% shade that were crossed to each other experienced a 79.9% reduction in egg hatch rate when crossed to each other relative to crosses with uninfected males (Mann-Whitney U: Z = 5.615, P < 0.001), suggesting a greatly reduced ability of females to restore compatibility under these rearing conditions.

**Fig 4:**
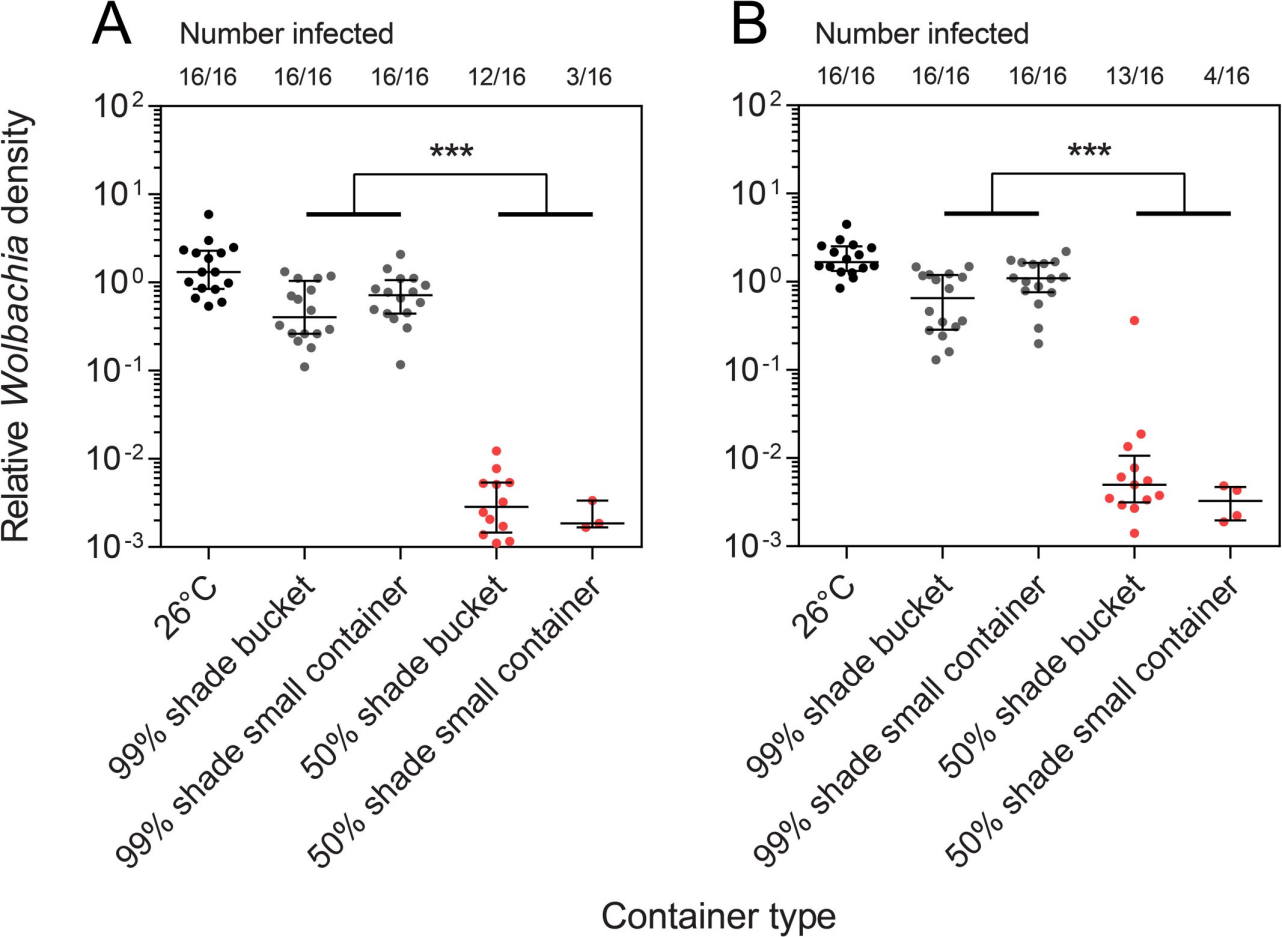
***Wolbachia* density of (A) Females and (B) males reared in different container types at two levels of shade in the second field experiment.** The number of mosquitoes from each treatment that were positive for *Wolbachia* is also shown. All error bars are medians with interquartile ranges. *** P < 0.001 by Kruskal-Wallis test.

We estimated the *Wolbachia* density of adults that emerged from each treatment in the second experiment. We found that many individuals had lost their *Wolbachia* infection (no detectable infection) when reared in containers held in 50% shade (Fig 4), particularly in small containers where larvae experienced higher maximum daily temperatures (Fig 3A). Of the adults reared at 50% shade that were still infected with *Wolbachia*, their density had been reduced to 0.19% and 0.23% of the 26°C control in females and males respectively. In this experiment, *Wolbachia* density was also reduced at 99% shade relative to the 26°C control (females: χ^2^ = 14.828, df = 1, P < 0.001, males: χ^2^ = 16.519, df = 1, P < 0.001), with densities being approximately 50% the level of the control.

**Fig 5:**
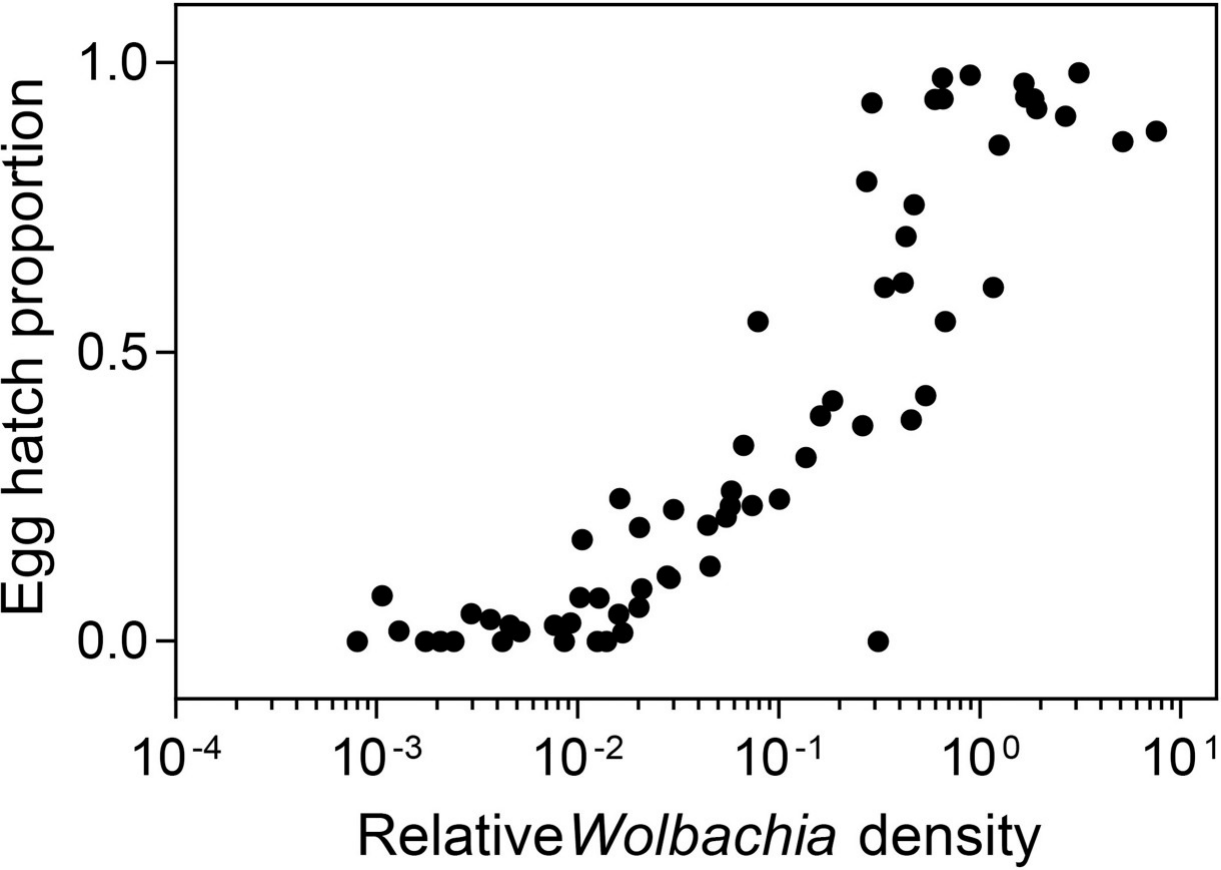
The relationship between female *Wolbachia* density and egg hatch proportion in crosses with *w*Mel-infected females and males reared under the same conditions in the field. Adults reared in buckets (8 L) or small containers (400 mL) held at 50% or 99% shade were crossed to individuals from the same rearing container before females were isolated for egg hatch rate and *Wolbachia* density measurements.

*w*Mel-infected adults emerging from the two container types and shade levels were returned to the laboratory and allowed to mate with individuals from the same container. We then scored egg hatch proportions of individual females and measured their *Wolbachia* density after oviposition to determine the relationship between *Wolbachia* density and egg hatch proportion. Females with high *Wolbachia* densities exhibited high hatch proportions while females with lower densities tended to have very low hatch proportions or produced no viable offspring, with the correlation between density and egg hatch being highly significant (Spearman’s rank-order correlation: ρ = 0.899, P < 0.001, n = 65, Fig 5). This indicates that females with low densities had partially or completely lost their ability to restore compatibility, but males reared under the same conditions had largely retained their ability to induce cytoplasmic incompatibility. The strong relationship between *Wolbachia* density and egg hatch indicates that a high density in females is important for restoring compatibility with infected males.

### Egg thermal tolerance and *Wolbachia* density

**Fig 6:**
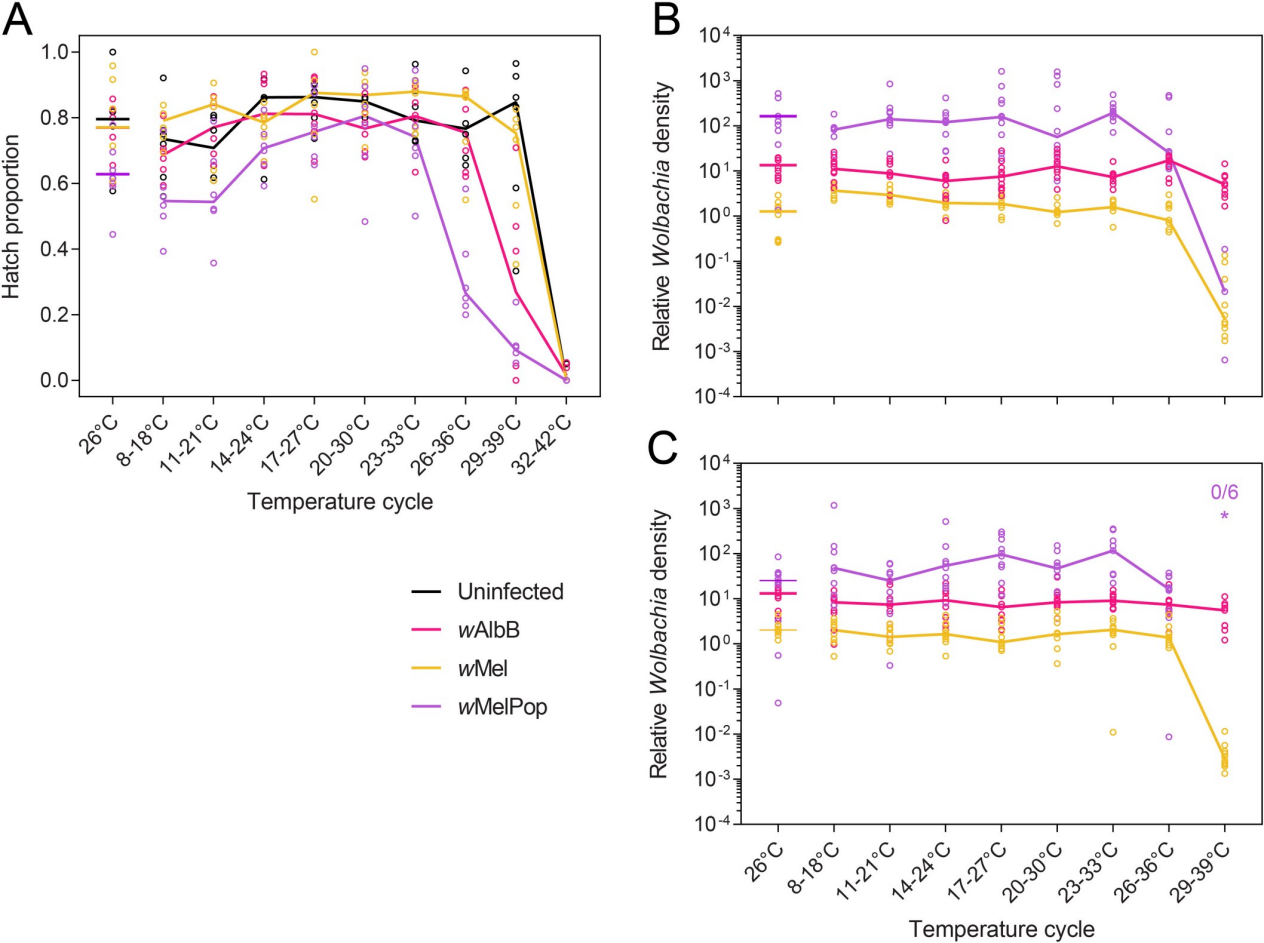
**Egg hatch proportions (A) and *Wolbachia* density in female (B) and male (C) adults after eggs were exposed to a broad range of temperature cycles for one week.** Each dot in (A) represents the proportion of eggs hatched in a replicate tube, with solid lines indicating median egg hatch proportions. Each dot in (B) and (C) represents the *Wolbachia* density of a single adult. Asterisks above temperature cycles indicate the number of individuals that were negative for *Wolbachia* out of the total number tested, with the color corresponding to the *Wolbachia* infection type.

We tested the tolerance of *Wolbachia*-infected and uninfected eggs to a broad range of temperature conditions. When eggs were held at 26°C for one week, *Wolbachia*-infected eggs did not differ from uninfected eggs in terms of hatch proportion (Mann-Whitney U: all P > 0.05). At higher temperatures fitness costs of *Wolbachia* infections were evident; *w*MelPop-infected eggs had lower hatch proportions than uninfected eggs under temperature cycles of 26–36°C and 29–39°C (both Z = 2.802, P = 0.005, Fig 6A). *w*AlbB-infected eggs also had reduced hatch proportions relative to uninfected eggs at 29–39°C (Z = 2.162, P = 0.031) but *w*Mel-infected eggs did not differ from uninfected eggs under any temperature cycle (all P > 0.05).

We reared larvae hatching from eggs held at each temperature cycle and measured *Wolbachia* density in adults. *Wolbachia* density did not differ between males and females across all temperature conditions (*w*Mel: Kruskal-Wallis χ^2^ = 0.271, df = 1, P = 0.603, *w*AlbB: χ^2^ = 2.398, df = 1, P = 0.122) except for *w*MelPop, where density was higher in females than in males (χ^2^ = 14.507, df = 1, P < 0.001). When eggs were held at 26°C, *w*MelPop-infected adults had the highest density of *Wolbachia* while *w*AlbB had an intermediate density and *w*Mel had the lowest density (Fig 6B and 6C), consistent with previous studies [20, 38]. This pattern was consistent across the cooler temperature cycles (maximum daily temperatures of 18–33°C) where *Wolbachia* densities for *w*AlbB (χ^2^ = 6.505, df = 5, P = 0.260), *w*MelPop (χ^2^ = 9.108, df = 5, P = 0.105) and *w*Mel males (χ^2^ = 2.950, df = 5, P = 0.708) were stable (Fig 6B and 6C). In contrast, *w*Mel density in females declined with increasing maximum temperatures across this range (χ^2^ = 27.190, df = 5, P < 0.001). When eggs were held at 29–39°C, adult *Wolbachia* density declined steeply for both *w*Mel and wMelPop infections (Fig 6B and 6C). Median *w*Mel densities were reduced to only 0.41% and 0.14% of densities at 26°C in females and males, respectively. In *w*MelPop the relative loss was even steeper, with females reared from eggs held at 29–39°C having just 0.02% the density of females at 26°C, while *Wolbachia* was not detected in males. *w*AlbB density also declined when eggs were held at 29–39°C in both females (Mann-Whitney U: Z = 2.797, P = 0.005) and males (Z = 2.735, P = 0.006) but the effect was much weaker than the other strains, with median densities of 34.88% (in females) and 42.04% (in males) of eggs held at 26°C.

**Fig 7:**
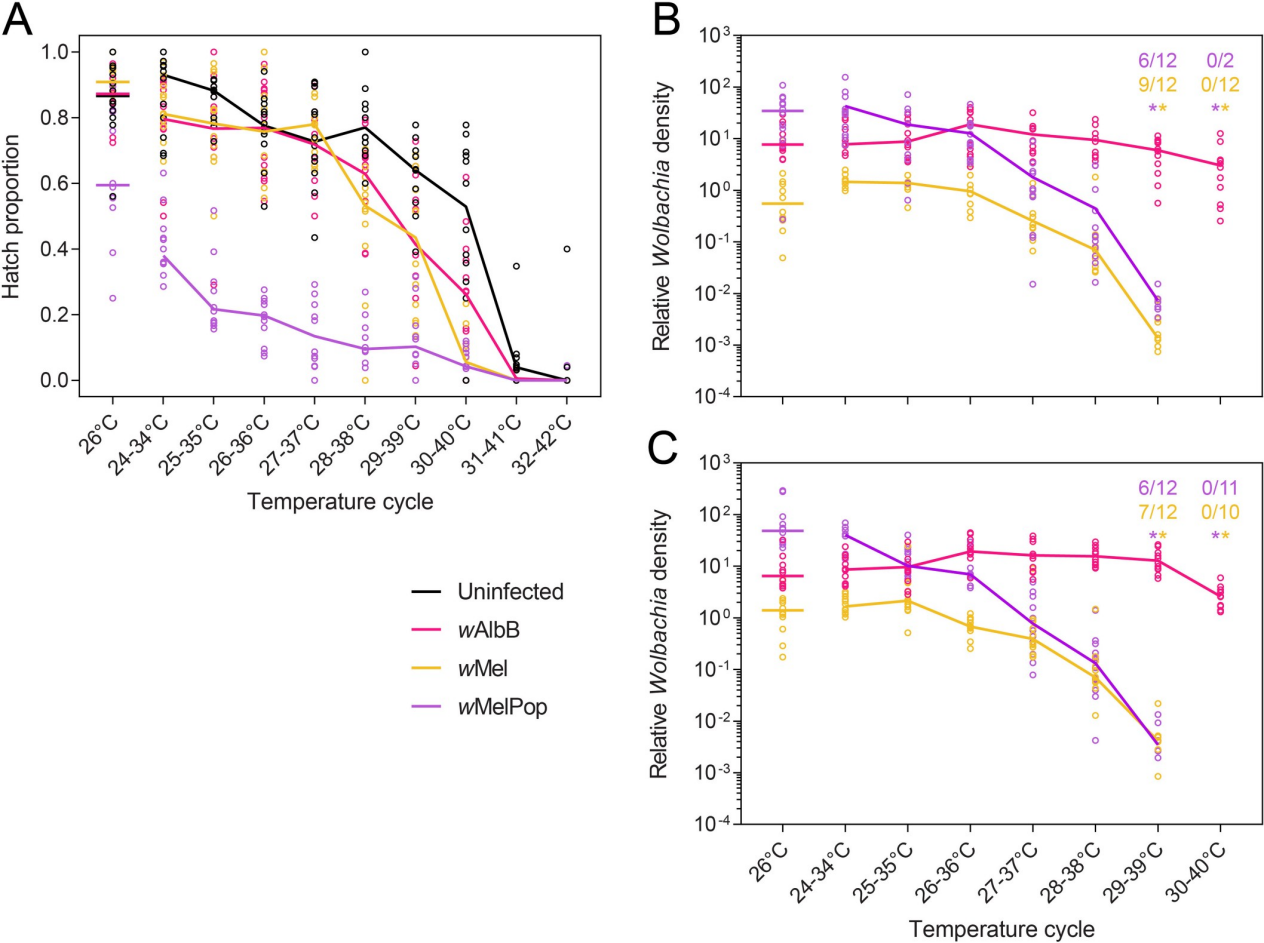
**Egg hatch proportions (A) and *Wolbachia* density in female (B) and male (C) adults after eggs were exposed to stressful temperature cycles for one week.** Each dot in (A) represents the proportion of eggs hatched in a replicate tube, with solid lines indicating median egg hatch proportions. Each dot in (B) and (C) represents the *Wolbachia* density of a single adult. Asterisks above temperature cycles indicate the number of individuals that were negative for *Wolbachia* out of the total number tested, with the color corresponding to the *Wolbachia* infection type.

In a second experiment, we used a narrower temperature range to investigate egg thermal tolerance and the loss of *Wolbachia* infections on a finer scale and with greater replication. Egg hatch proportions declined for all *Wolbachia* infection types as maximum temperatures increased, with the effect being most severe for the *w*MelPop infection (Fig 7A). Egg hatch proportions of the *w*Mel and *w*AlbB strains did not differ significantly from uninfected eggs at maximum temperatures of 37°C and below (Mann-Whitney U: all P > 0.05). However, at maximum temperatures of 38–41°C both the wMel and wAlbB strains had lower hatch proportions than uninfected eggs held at the same temperature (all P ≤ 0.026). This indicates that *Wolbachia* infections in *Ae*. *aegypti* lower the tolerance of eggs to high temperatures, particularly in the case of *w*MelPop.

Consistent with the previous experiment, *Wolbachia* density declined as eggs were exposed to increasing maximum temperatures, beginning at 35°C for *w*MelPop and 36°C for *w*Mel (Fig 7B and 7C). The *w*Mel and *w*MelPop infections were lost from some individuals when eggs were exposed to 29–39°C and absent from all adults at 30–40°C (Fig 7B and 7C). In contrast, all *w*AlbB adults were infected across all temperature cycles, though density was reduced in both females (Mann-Whitney U: Z = 3.204, P = 0.001) and males (Z = 3.897, P < 0.001) at 30–40°C relative to 26°C.

## Discussion

We tested the stability of the *w*Mel *Wolbachia* infection in *Ae*. *aegypti* under field temperature conditions and performed laboratory experiments to determine the range of temperatures that affect different *Wolbachia* strains. Our experiments demonstrate three main outcomes of heat stress on *Wolbachia*-infected mosquitoes. Firstly, there are direct costs of *Wolbachia* infections on *Ae*. *aegypti* thermal tolerance, at least during the egg stage. Secondly, heat stress under partial shade conditions in the field reduces cytoplasmic incompatibility fidelity in *w*Mel-infected males, while infected females become partially incompatible with infected males. Thirdly, heat stress reduces *Wolbachia* density and may impair the ability of *Wolbachia* to block virus transmission for a subsection of the mosquito population reared under specific field conditions. Heat stress could therefore adversely affect the success of disease control programs depending on the location and nature of the field breeding sites.

There are relatively few examples of symbionts affecting the thermal tolerance of their hosts [48]. In *Drosophila melanogaster*, the *w*MelCS strain of *Wolbachia* increases the survival of adults under heat stress while the *w*MelPop infection decreases survival [49], though *w*Mel appears to have no effect on high temperature tolerance [50]. Here we show that *Wolbachia* infection reduces the tolerance of *Ae*. *aegypti* eggs to high temperatures, with the severity of the effect depending on *Wolbachia* strain. In addition, we have determined the temperature range where deleterious effects on *Wolbachia* infections start to occur and where the infections are lost, at least during the egg stage. For the *w*MelPop and *w*Mel infections, *Wolbachia* density declined beginning at temperatures of 25–35°C (30°C mean) and 26–36°C (31°C mean) respectively, while for *w*AlbB this occurred at a much higher temperature range (30–40°C, 35°C mean). The higher tolerance of *w*AlbB to heat stress is consistent with prior studies in *Ae*. *aegypti* larvae [19, 35, 38], but the increased resolution in this experiment provides a better estimate of the maximum daily temperatures that could affect *Wolbachia* interventions. In field situations the temperature ranges where *Wolbachia* infections are adversely affected will depend on the duration and timing of heat stress (see below).

In our field experiments we found substantial effects of heat stress on cytoplasmic incompatibility that could limit the potential of *w*Mel to invade natural populations during disease control programs and persist following releases. When reared in partial shade, *w*Mel-infected males partially lost their ability to induce cytoplasmic incompatibility while females partially or completely lost their ability to restore compatibility when crossed to infected males reared in the lab. Infected females reared in partial shade had greatly reduced fertility in crosses with infected males from the same container. Female *Wolbachia* density was positively associated with egg hatch, consistent with a study in *Drosophila* [51]. High densities therefore appear needed for females to restore compatibility with infected males, but the density required for males to induce cytoplasmic incompatibility appears to be lower. Heat stress conditions in the field could greatly diminish or even reverse the reproductive advantage provided by *Wolbachia*, making invasion challenging, particularly when *Wolbachia* is at a low frequency, when its fitness relative to uninfected individuals is relatively lower and where it is susceptible to stochastic [52, 53] and density related [30, 54] effects. Where *w*Mel has already established in a population, reduced egg hatch in *w*Mel-infected mosquitoes that mate with each other could provide an opportunity for an increase in the frequency of uninfected mosquitoes although once *w*Mel invaded areas of North Queensland it appears to have been stable [34]. Fitness costs and self-incompatibility between infected mosquitoes could also have unexpected ecological effects; a decline in the *Ae*. *aegypti* population could lead to shifts in species composition [24] which could be beneficial for disease control efforts.

Though we attempted to rear mosquitoes under realistic temperature conditions, our field experiments will only be relevant to a subset of natural breeding sites. We provided abundant food to speed up and synchronise larval development to facilitate experimental crosses between strains. Larval development times in nature are variable and can exceed two months under competitive conditions [30]. Increasing the rate of larval development in this experiment likely underestimated the effect of heat stress; longer development times increase the chance that larvae will experience a heat wave and increased durations of heat stress may further reduce *Wolbachia* density, though density may also recover over time in the absence of heat stress [37]. *w*Mel-infected larvae provided with a low level of food have a greatly reduced *Wolbachia* density when reared at 26–32°C compared to 26°C, indicating that even moderate temperatures can reduce *Wolbachia* density when combined with nutritional stress (see Figure S4 of Ross and Hoffmann [35]). The effects of heat stress on *Wolbachia* density can carry over into the next generation [55] which may lead to reduced virus blockage or cytoplasmic incompatibility across a generation after a heat wave. In our laboratory experiments eggs were maintained for one week before hatching, but in the field the egg stage can be shorter or much longer. During the dry season eggs can remain quiescent for months before hatching [56], increasing their potential exposure to high temperatures. *Wolbachia* infections reduce the viability of quiescent Ae. *aegypti* eggs [20, 26, 27] and under high temperatures these fitness costs will likely be exacerbated.

A further limitation of our field experiment is that the containers used for larval rearing were not colonized naturally. *Aedes aegypti* seem to prefer laying eggs in shaded areas but will also utilize containers in sunlight [57–59]. *Wolbachia* infections may also affect thermal preference; adult *Drosophila melanogaster* infected with *Wolbachia* prefer cooler temperatures than uninfected flies [60, 61]. Nevertheless, data from sentinel containers indicates that *w*Mel-infected mosquitoes will lay eggs in containers placed in direct sunlight. Sentinel buckets and small containers placed within the *w*Mel release zone in Cairns were all colonized by *Ae*. *aegypti* despite some of these experiencing similar temperatures to the experimental containers held at 50% shade (S2 Fig). *Ae*. *aegypti* tend to lay eggs during cooler parts of the day [62, 63] and therefore may be unable to discriminate against habitats that reach high maximum temperatures later. Unlike adult mosquitoes, immature stages cannot easily escape heat stress as they are unable to move beyond the container. Since *Wolbachia* density and egg hatch in *w*Mel-infected mosquitoes appears to depend strongly on the level of shade, temperature and productivity surveys of larval habitats could be conducted in release areas if there are concerns around heat stress impacts in a release area.

Despite the substantial effects on *Wolbachia* density and fertility in our experiments, *Ae*. *aegypti* mosquitoes infected with *w*Mel have successfully established in Cairns [11, 12] and Townsville [13], Australia and in Brazil [23], with the infection persisting at a high frequency in most locations. In areas where the releases succeeded, the costs of heat stress observed here were clearly not prevalent or severe enough to prevent the establishment of *w*Mel. Once a *Wolbachia* infection has attained a high frequency in a population it may stay high unless the fitness costs are extreme, as is the case for *w*MelPop [28]. Nevertheless, heat stress will likely slow the rate of *Wolbachia* invasion and spread, increasing the number of mosquitoes required for releases, and potentially creating an unstable situation around critical invasion points that must be exceeded for *Wolbachia* to invade [52]. Heat stress could partially explain why infection frequencies have persisted at intermediate levels in some suburbs [13] and may also contribute to the incomplete maternal transmission fidelity of *w*Mel observed in Cairns [64] given that some individuals were cleared of their *Wolbachia* infections in our experiments. *w*Mel-infected mosquito releases outside of Australia in locations where maximum daily temperatures are warmer may be more challenging. Reduced *Wolbachia* densities may also reduce virus protection provided by *Wolbachia* even if infection frequencies remain high in a population, though we do not demonstrate this effect directly. The *w*Mel strain has retained its susceptibility to heat stress for seven years after field deployment in Australia [35], indicating that alternative strains may be needed in areas where *w*Mel has difficulty establishing or where viral blockage is insufficient.

## Supporting information

S1 Data All temperature, hatch proportion and *Wolbachia* density data for experiments. (XLSX)

S1 Fig Temperature cycles experienced in buckets (solid lines), plant pots (dashed lines) and cups (dotted lines) in 99% shade (black lines) and 50% shade (red lines) during the first field experiment from the 10th to the 17th of January 2018. (EPS)

S2 Fig Temperatures in sentinel containers placed within the *w*Mel release zone in suburban Cairns. (A) Temperatures recorded by data loggers placed in sentinel buckets (solid lines) and small containers (dashed lines). Containers were either left in the open (red lines), placed in a garden bed (blue lines) or placed under cover (black lines). All containers were positive for *Ae*. *aegypti* eggs within one week of placement, and temperatures are only shown for the period after containers were confirmed as *Ae*. *aegypti* larval habitats. (B) Comparison of temperatures in buckets and small containers used in the second field experiment (red lines) and uncovered sentinel containers (black lines) recorded during the same period.
(EPS)

S3 Fig Temperature cycles of thermocyclers in the first (A) and second (B) egg thermal tolerance experiment.
(EPS)

## References

[1] Salazar MI, Richardson JH, Sanchez-Vargas I, Olson KE, Beaty BJ. Dengue virus type 2: replication and tropisms in orally infected *Aedes aegypti* mosquitoes. BMC microbiol. 2007;7:9 10.1186/1471-2180-7-9 17263893 PMC1797809

[2] Kraemer MU, Sinka ME, Duda KA, Mylne AQ, Shearer FM, Barker CM, et al The global distribution of the arbovirus vectors *Aedes aegypti* and *Ae*. *albopictus*. eLife. 2015;4 10.7554/eLife.08347 . 26126267 PMC4493616

[3] Endersby-Harshman NM, Wuliandari JR, Harshman LG, Frohn V, Johnson BJ, Ritchie SA, et al Pyrethroid susceptibility has been maintained in the dengue vector, *Aedes aegypti* (Diptera: Culicidae), in Queensland, Australia. J Med Ent. 2017 10.1093/jme/tjx145 28981684

[4] Moyes CL, Vontas J, Martins AJ, Ng LC, Koou SY, Dusfour I, et al Contemporary status of insecticide resistance in the major Aedes vectors of arboviruses infecting humans. PLoS Negl Trop Dis. 2017;11(7):e0005625 Epub 2017/07/21. 10.1371/journal.pntd.0005625 28727779 PMC5518996

[5] Ritchie SA, Johnson BJ. Advances in vector control science: rear-and-release strategies show promise … but don't forget the basics. J Infect Dis. 2017;215(suppl_2):S103–S8. 10.1093/infdis/jiw575 . 28403439

[6] Moreira LA, Iturbe-Ormaetxe I, Jeffery JA, Lu G, Pyke AT, Hedges LM, et al A *Wolbachia* symbiont in *Aedes aegypti* limits infection with dengue, Chikungunya, and Plasmodium. Cell. 2009;139(7):1268–78. 10.1016/j.cell.2009.11.042 . 20064373

[7] van den Hurk AF, Hall-Mendelin S, Pyke AT, Frentiu FD, McElroy K, Day A, et al Impact of *Wolbachia* on infection with chikungunya and yellow fever viruses in the mosquito vector *Aedes aegypti*. PLoS Negl Trop Dis. 2012;6(11):e1892 10.1371/journal.pntd.0001892 23133693 PMC3486898

[8] Ye YH, Woolfit M, Rances E, O'Neill SL, McGraw EA. *Wolbachia*-associated bacterial protection in the mosquito *Aedes aegypti*. PLoS Negl Trop Dis. 2013;7(8):e2362 10.1371/journal.pntd.0002362 23951381 PMC3738474

[9] O'Neill SL, Hoffmann AA, Werren JH. Influential passengers: inherited microorganisms and arthropod reproduction: Oxford University Press Oxford; 1997.

[10] Zug R, Hammerstein P. Bad guys turned nice? A critical assessment of *Wolbachia* mutualisms in arthropod hosts. Biol Rev Camb Philos Soc. 2014 10.1111/brv.12098 . 24618033

[11] Hoffmann AA, Montgomery BL, Popovici J, Iturbe-Ormaetxe I, Johnson PH, Muzzi F, et al Successful establishment of *Wolbachia* in *Aedes* populations to suppress dengue transmission. Nature. 2011;476(7361):454–7. http://www.nature.com/nature/journal/v476/n7361/abs/nature10356.html#supplementary-information. 10.1038/nature10356 21866160

[12] Schmidt TL, Barton NH, Rasic G, Turley AP, Montgomery BL, Iturbe-Ormaetxe I, et al Local introduction and heterogeneous spatial spread of dengue-suppressing *Wolbachia* through an urban population of *Aedes aegypti*. PLoS Biol. 2017;15(5):e2001894 10.1371/journal.pbio.2001894 . 28557993 PMC5448718

[13] O'Neill SL, Ryan PA, Turley AP, Wilson G, Retzki K, Iturbe-Ormaetxe I, et al Scaled deployment of *Wolbachia* to protect the community from dengue and other *Aedes* transmitted arboviruses. Gates Open Res. 2018;2. 10.12688/gatesopenres.12844.2 PMC6305154 30596205

[14] O'Connor L, Plichart C, Sang AC, Brelsfoard CL, Bossin HC, Dobson SL. Open release of male mosquitoes infected with a *Wolbachia* biopesticide: field performance and infection containment. PLoS Negl Trop Dis. 2012;6(11):e1797 10.1371/journal.pntd.0001797 23166845 PMC3499408

[15] Mains JW, Brelsfoard CL, Rose RI, Dobson SL. Female adult *Aedes albopictus* suppression by *Wolbachia*-infected male mosquitoes. Sci Rep. 2016;6:33846 10.1038/srep33846 27659038 PMC5034338

[16] Xi Z, Khoo CC, Dobson SL. *Wolbachia* establishment and invasion in an *Aedes aegypti* laboratory population. Science. 2005;310(5746):326–8. 10.1126/science.1117607 . 16224027

[17] McMeniman CJ, Lane RV, Cass BN, Fong AW, Sidhu M, Wang Y-F, et al Stable introduction of a life-shortening *Wolbachia* infection into the mosquito *Aedes aegypti*. Science. 2009;323(5910):141–4. 10.1126/science.1165326 19119237

[18] Fraser JE, De Bruyne JT, Iturbe-Ormaetxe I, Stepnell J, Burns RL, Flores HA, et al Novel *Wolbachia*-transinfected *Aedes aegypti* mosquitoes possess diverse fitness and vector competence phenotypes. PLoS Pathog. 2017;13(12):e1006751 Epub 2017/12/08. 10.1371/journal.ppat.1006751 . 29216317 PMC5736235

[19] Ant TH, Herd CS, Geoghegan V, Hoffmann AA, Sinkins SP. The *Wolbachia* strain *w*Au provides highly efficient virus transmission blocking in *Aedes aegypti*. PLoS Pathog. 2018;14(1):e1006815 Epub 2018/01/26. 10.1371/journal.ppat.1006815 . 29370307 PMC5784998

[20] Axford JK, Ross PA, Yeap HL, Callahan AG, Hoffmann AA. Fitness of *w*AlbB *Wolbachia* infection in *Aedes aegypti*: parameter estimates in an outcrossed background and potential for population invasion. Am J Trop Med Hyg. 2016;94(3):507–16. 10.4269/ajtmh.15-0608 . 26711515 PMC4775882

[21] Joubert DA, Walker T, Carrington LB, De Bruyne JT, Kien DH, Hoang Nle T, et al Establishment of a *Wolbachia* superinfection in *Aedes aegypti* mosquitoes as a potential approach for future resistance management. PLoS Pathog. 2016;12(2):e1005434 10.1371/journal.ppat.1005434 26891349 PMC4758728

[22] Ant TH, Sinkins SP. A *Wolbachia* triple-strain infection generates self-incompatibility in *Aedes albopictus* and transmission instability in *Aedes aegypti*. Parasit Vectors. 2018;11(1):295 Epub 2018/05/13. 10.1186/s13071-018-2870-0 29751814 PMC5948879

[23] Garcia GdA, Sylvestre G, Aguiar R, da Costa GB, Martins AJ, Lima JBP, et al Matching the genetics of released and local *Aedes aegypti* populations is critical to assure *Wolbachia* invasion. PLoS Negl Trop Dis. 2019;13(1):e0007023 10.1371/journal.pntd.0007023 30620733 PMC6338382

[24] Ritchie SA, van den Hurk AF, Smout MJ, Staunton KM, Hoffmann AA. Mission accomplished? We need a guide to the ‘post release’ world of *Wolbachia* for *Aedes*-borne disease control. Trends Parasitol. 2018 10.1016/j.pt.2017.11.011 29396201

[25] Ross PA, Endersby NM, Hoffmann AA. Costs of three *Wolbachia* infections on the survival of *Aedes aegypti* larvae under starvation conditions. PLoS Negl Trop Dis. 2016;10(1):e0004320 10.1371/journal.pntd.0004320 26745630 PMC4706305

[26] McMeniman CJ, O'Neill SL. A virulent *Wolbachia* infection decreases the viability of the dengue vector *Aedes aegypti* during periods of embryonic quiescence. PLoS Negl Trop Dis. 2010;4(7):e748 10.1371/journal.pntd.0000748 20644622 PMC2903475

[27] Yeap HL, Mee P, Walker T, Weeks AR, O'Neill SL, Johnson P, et al Dynamics of the "popcorn" *Wolbachia* infection in outbred *Aedes aegypti* informs prospects for mosquito vector control. Genetics. 2011;187(2):583–95. 10.1534/genetics.110.122390 21135075 PMC3030498

[28] Nguyen TH, Nguyen HL, Nguyen TY, Vu SN, Tran ND, Le TN, et al Field evaluation of the establishment potential of *w*MelPop *Wolbachia* in Australia and Vietnam for dengue control. Parasit Vectors. 2015;8:563 10.1186/s13071-015-1174-x 26510523 PMC4625535

[29] Calvitti M, Marini F, Desiderio A, Puggioli A, Moretti R. *Wolbachia* density and cytoplasmic incompatibility in *Aedes albopictus*: concerns with using artificial *Wolbachia* infection as a vector suppression tool. PloS One. 2015;10(3):e0121813 10.1371/journal.pone.0121813 25812130 PMC4374832

[30] Hancock PA, Linley-White V, Callahan AG, Godfray HCJ, Hoffmann AA, Ritchie SA. Density-dependent population dynamics in *Aedes aegypti* slow the spread of *w*Mel Wolbachia. J Appl Ecol. 2016:n/a-n/a. 10.1111/1365-2664.12620

[31] Hancock PA, Ritchie SA, Koenraadt CJ, Scott TW, Hoffmann AA, Godfray HCJ. Predicting the spatial dynamics of *Wolbachia* infections in *Aedes aegypti* arbovirus vector populations in heterogeneous landscapes. bioRxiv. 2018:458794.

[32] Bull JJ, Turelli M. *Wolbachia* versus dengue: Evolutionary forecasts. Evol Med Public Health. 2013;2013(1):197–207. 10.1093/emph/eot018 24481199 PMC3847891

[33] Frentiu FD, Zakir T, Walker T, Popovici J, Pyke AT, van den Hurk A, et al Limited dengue virus replication in field-collected *Aedes aegypti* mosquitoes infected with *Wolbachia*. PLoS Negl Trop Dis. 2014;8(2):e2688 10.1371/journal.pntd.0002688 24587459 PMC3930499

[34] Hoffmann AA, Iturbe-Ormaetxe I, Callahan AG, Phillips BL, Billington K, Axford JK, et al Stability of the *w*Mel *Wolbachia* infection following invasion into *Aedes aegypti* populations. PLoS Negl Trop Dis. 2014;8(9):e3115 10.1371/journal.pntd.0003115 . 25211492 PMC4161343

[35] Ross PA, Hoffmann AA. Continued susceptibility of the *w*Mel *Wolbachia* infection in *Aedes aegypti* to heat stress following field deployment and selection. Insects. 2018;9(3). Epub 2018/07/04. 10.3390/insects9030078 29966368 PMC6165456

[36] Scheffers BR, De Meester L, Bridge TC, Hoffmann AA, Pandolfi JM, Corlett RT, et al The broad footprint of climate change from genes to biomes to people. Science. 2016;354(6313):aaf7671 10.1126/science.aaf7671 27846577

[37] Ulrich JN, Beier JC, Devine GJ, Hugo LE. Heat sensitivity of *w*Mel *Wolbachia* during *Aedes aegypti* development. PLoS Negl Trop Dis. 2016;10(7):e0004873 10.1371/journal.pntd.0004873 27459519 PMC4961373

[38] Ross PA, Wiwatanaratanabutr I, Axford JK, White VL, Endersby-Harshman NM, Hoffmann AA. *Wolbachia* infections in *Aedes aegypti* differ markedly in their response to cyclical heat stress. PLoS Pathog. 2017;13(1):e1006006 10.1371/journal.ppat.1006006 28056065 PMC5215852

[39] Hoffmann AA, Turell MJ. Cytoplasmic incompatibility in insects. Influential Passengers: Inherited Microorganisms and Arthropod Reproduction 1997 p. 42–80.

[40] Osborne SE, Iturbe-Ormaetxe I, Brownlie JC, O'Neill SL, Johnson KN. Antiviral protection and the importance of *Wolbachia* density and tissue tropism in *Drosophila simulans*. Appl Environ Microbiol. 2012;78(19):6922–9. 10.1128/AEM.01727-12 22843518 PMC3457512

[41] Martinez J, Tolosana I, Ok S, Smith S, Snoeck K, Day JP, et al Symbiont strain is the main determinant of variation in *Wolbachia*-mediated protection against viruses across *Drosophila* species. Mol Ecol. 2017 10.1111/mec.14164 . 28464440 PMC5966720

[42] Lu P, Bian G, Pan X, Xi Z. *Wolbachia* induces density-dependent inhibition to dengue virus in mosquito cells. PLoS Negl Trop Dis. 2012;6(7):e1754 10.1371/journal.pntd.0001754 22848774 PMC3404113

[43] Ross PA, Axford JK, Richardson KM, Endersby-Harshman NM, Hoffmann AA. Maintaining *Aedes aegypti* mosquitoes infected with *Wolbachia*. J Vis Exp. 2017;(126). 10.3791/56124 28829414 PMC5614331

[44] Ritchie SA, Townsend M, Paton CJ, Callahan AG, Hoffmann AA. Application of *w*MelPop *Wolbachia* strain to crash local populations of *Aedes aegypti*. PLoS Negl Trop Dis. 2015;9(7):e0003930 10.1371/journal.pntd.0003930 26204449 PMC4512704

[45] Kong JD, Axford JK, Hoffmann AA, Kearney MR. Novel applications of thermocyclers for phenotyping invertebrate thermal responses. Methods Ecol Evol. 2016 10.1111/2041-210x.12589

[46] Ross PA, Endersby NM, Yeap HL, Hoffmann AA. Larval competition extends developmental time and decreases adult size of *w*MelPop *Wolbachia*-infected *Aedes aegypti*. Am J Trop Med Hyg. 2014;91(1):198–205. 10.4269/ajtmh.13-0576 24732463 PMC4080562

[47] Lee SF, White VL, Weeks AR, Hoffmann AA, Endersby NM. High-throughput PCR assays to monitor *Wolbachia* infection in the dengue mosquito (*Aedes aegypti*) and *Drosophila simulans*. Appl Environ Microbiol. 2012;78(13):4740–3. 10.1128/AEM.00069-12 22522691 PMC3370494

[48] Corbin C, Heyworth ER, Ferrari J, Hurst GDD. Heritable symbionts in a world of varying temperature. Heredity. 2017;118(1):10–20. 10.1038/hdy.2016.71 27703153 PMC5176117

[49] Gruntenko NE, Ilinsky YY, Adonyeva NV, Burdina EV, Bykov RA, Menshanov PN, et al Various *Wolbachia* genotypes differently influence host *Drosophila* dopamine metabolism and survival under heat stress conditions. BMC Evol Biol. 2017;17(Suppl 2):252 Epub 2018/01/04. 10.1186/s12862-017-1104-y 29297293 PMC5751659

[50] Rolandi C, Lighton JRB, de la Vega GJ, Schilman PE, Mensch J. Genetic variation for tolerance to high temperatures in a population of *Drosophila melanogaster*. Ecol Evol. 2018;8(21):10374–83. Epub 2018/11/23. 10.1002/ece3.4409 30464811 PMC6238130

[51] Clancy DJ, Hoffmann AA. Environmental effects on cytoplasmic incompatibility and bacterial load in *Wolbachia*‐infected *Drosophila simulans*. Entomol Exp Appl. 1998;86(1):13–24.

[52] Jansen VA, Turelli M, Godfray HC. Stochastic spread of *Wolbachia*. Proc R Soc B. 2008;275(1652):2769–76. 10.1098/rspb.2008.0914 18755670 PMC2605827

[53] Hu L, Huang M, Tang M, Yu J, Zheng B. *Wolbachia* spread dynamics in stochastic environments. Theor Popul Biol. 2015 10.1016/j.tpb.2015.09.003 . 26428255

[54] Hancock PA, Sinkins SP, Godfray HC. Population dynamic models of the spread of *Wolbachia*. Am Nat. 2011;177(3):323–33. 10.1086/658121 . 21460541

[55] Foo IJH, Hoffmann AA, Ross PA. Cross-generational effects of heat stress on fitness and *Wolbachia* density in *Aedes aegypti* mosquitoes. Trop Med Infect Dis. 2019;4(1):13. 10.3390/tropicalmed4010013 PMC6473245 30642130

[56] Faull KJ, Williams CR. Intraspecific variation in desiccation survival time of *Aedes aegypti* (L.) mosquito eggs of Australian origin. J Vector Ecol. 2015;40(2):292–300. 10.1111/jvec.12167 26611964

[57] Tun-Lin W, Kay B, Barnes A. The premise condition index: a tool for streamlining surveys of *Aedes aegypti*. Am J Trop Med Hyg. 1995;53(6):591–4. 8561259 10.4269/ajtmh.1995.53.591

[58] Maciel-de-Freitas R, Marques WA, Peres RC, Cunha SP, Lourenço-de-Oliveira R. Variation in *Aedes aegypti* (Diptera: Culicidae) container productivity in a slum and a suburban district of Rio de Janeiro during dry and wet seasons. Mem Inst Oswaldo Cruz. 2007;102(4):489–96. 17612770 10.1590/s0074-02762007005000056

[59] Vezzani D, Albicocco A. The effect of shade on the container index and pupal productivity of the mosquitoes *Aedes aegypti* and *Culex pipiens* breeding in artificial containers. Med Vet Entomol. 2009;23(1):78–84. 10.1111/j.1365-2915.2008.00783.x 19239617

[60] Arnold PA, Levin SC, Stevanovic AL, Johnson KN. *Drosophila melanogaster* infected with Wolbachia strain *w*MelCS prefer cooler temperatures. Ecol Entomol. 2018.

[61] Truitt AM, Kapun M, Kaur R, Miller WJ. *Wolbachia* modifies thermal preference in *Drosophila melanogaster*. Environ Microbiol. 2018 Epub 2018/07/05. 10.1111/1462-2920.14347 . 29971900 PMC6766989

[62] Corbet PS, Chadee DD. Incidence and diel pattern of oviposition outdoors of the mosquito, *Aedes aegypti* (L.)(Diptera: Culicidae) in Trinidad, WI in relation to solar aspect. Ann Trop Med Parasitol. 1990;84(1):63–78. 2331177 10.1080/00034983.1990.11812434

[63] Harrington LC, Ponlawat A, Edman JD, Scott TW, Vermeylen F. Influence of container size, location, and time of day on oviposition patterns of the dengue vector, *Aedes aegypti*, in Thailand. Vector Borne and Zoonotic Dis. 2008;8(3):415–23. 10.1089/vbz.2007.0203 18279006 PMC2978047

[64] Schmidt TL, Filipović I, Hoffmann AA, Rašić G. Fine-scale landscape genomics helps explain the slow spatial spread of *Wolbachia* through the *Aedes aegypti* population in Cairns, Australia. Heredity. 2018 10.1038/s41437-017-0039-9 29358725 PMC5889405

